# Evaluating Family Coping Mechanisms in Pediatric Seizure Disorders: From Emergency Room to Long-Term Follow-Up

**DOI:** 10.3390/pediatric16030055

**Published:** 2024-07-31

**Authors:** Ada Claudia Silvana Gruescu, Calin Popoiu, Mihaela Codrina Levai, Paula Irina Barata, Caius Glad Streian

**Affiliations:** 1 Department of Pediatrics, Victor Babes University of Medicine and Pharmacy Timisoara, 300041 Timisoara, Romania; ada.gruescu@umft.ro (A.C.S.G.); mcpopoiu@umft.ro (C.P.); 2 Doctoral School, Victor Babes University of Medicine and Pharmacy Timisoara, 300041 Timisoara, Romania; 3 Research Center for Medical Communication, Victor Babes University of Medicine and Pharmacy Timisoara, 300041 Timisoara, Romania; codrinalevai@umft.ro; 4 Center for Research and Innovation in Precision Medicine of Respiratory Diseases, University of Medicine and Pharmacy Victor Babes Timisoara, 300041 Timisoara, Romania; 5 Department of Physiology, Faculty of Medicine, “Vasile Goldis” Western University of Arad, 310025 Arad, Romania; 6 Department of Cardiac Surgery, “Victor Babes” University of Medicine and Pharmacy Timisoara, Eftimie Murgu sq, 300041 Timisoara, Romania; streian.caius@umft.ro

**Keywords:** child neurology, parental stress, anxiety, depression, coping strategies

## Abstract

Pediatric seizure disorders profoundly impact family dynamics, often escalating stress and impairing coping mechanisms. This study aimed to longitudinally assess the impact of pediatric seizures on family stress and coping, evaluating the efficacy of multidisciplinary follow-up care in enhancing psychological resilience and adaptation. A longitudinal study design was implemented, enrolling children aged 1–18 who presented with a first seizure and received a neurologist’s diagnosis at the Emergency Clinical Hospital for Children “Louis Turcanu,” Timisoara, Romania. Validated questionnaires, including the Parenting Stress Index (PSI), Hospital Anxiety and Depression Scale (HADS), Perceived Stress Scale (PSS-10), and Parental Concerns Questionnaire (PCQ), were employed at baseline, 6, and 12 months. Statistical analysis utilized ANOVA and t-tests to evaluate changes in stress and coping outcomes. The study involved 68 families, with significant reductions in stress and anxiety reported over the follow-up period. Initial PSI scores showed high stress levels across various domains: Emotional Stress (59.47) decreased to 50.63 at one year; Parent–Child Communication Difficulties started at 66.03 ± 20.15 and reduced to 56.92 ± 18.74; and Total Family Stress decreased from 65.55 to 55.97. The HADS scores indicated initial anxiety and depression at 8.2 ± 3.1 and 7.1 ± 2.8, respectively, with reductions to 6.8 and 5.9 by the end of the year. The overall HADS score showed a decrease from 15.4 to 12.8. PCQ results mirrored these findings, with Total Score dropping from 9.7 to 7.7. PSS-10 scores declined from 13.5 to 11.3, with a significant reduction in the positive sub-score. The proactive, multidisciplinary care approach significantly reduced stress and enhanced coping mechanisms in families dealing with pediatric seizures. The decreases in stress, anxiety, and depression scores highlight the potential for integrated care models to improve long-term outcomes in these families. These findings support the continued development of targeted interventions to aid in the management of chronic pediatric conditions.

## 1. Introduction

Seizure disorders are among the most common neurological conditions encountered in pediatric populations, significantly impacting patients and their families [1,2]. Emergency room visits for initial seizure episodes in children not only trigger an acute medical response but also initiate a long-term psychological and social challenge for their families [3]. Research indicates that approximately 4–10 per 1000 children experience an unprovoked seizure, with a significant percentage developing recurrent seizures, thereby transitioning from acute to chronic management [4,5]. The initial emergency response and the subsequent uncertainty regarding seizure recurrence can profoundly affect family dynamics and coping strategies [6].

The psychological impact on families of children with seizure disorders is profound. Studies have shown that caregivers of children with chronic health conditions exhibit higher levels of stress, anxiety, and depression compared to the general population [7,8,9]. In the context of seizures, stress is marked by anticipation, being exacerbated by the unpredictable nature of seizures, the fear of seizure recurrence, and the potential for sudden deterioration [10,11]. The emergency department (ED) visit often marks the beginning of an ongoing emotional and logistical adjustment as families transition to managing a potentially long-term condition.

Despite the recognition of these challenges, there is a paucity of longitudinal data specifically addressing how families adapt over time to the diagnosis of a pediatric seizure disorder. Moreover, the role of interdisciplinary support in acute care settings holds a continuous research focus [12,13,14]. Pediatric neurology, psychology, social work, and nursing each play crucial roles in supporting families during this challenging time. Integrated care models that include regular follow-ups and psychological support have been suggested to improve outcomes, yet comprehensive models integrating these multiple perspectives are not widely implemented [15].

Furthermore, socioeconomic factors and access to healthcare resources also significantly influence stress levels and coping mechanisms in families dealing with pediatric seizure disorders [16]. Research has demonstrated disparities in how different populations access and utilize healthcare services, which can affect the management outcomes and stress levels experienced by families [17,18]. These disparities necessitate a deeper examination of how various demographic factors, such as those expressed by the Romanian population in the current economical context, influence family experiences and outcomes in the context of pediatric seizures [19,20,21].

Based on the gaps identified in the literature, this study hypothesizes that proactive, multidisciplinary follow-up care can significantly alleviate stress and improve coping mechanisms in families of children with pediatric seizure disorders. As the first follow-up study of its type developed in Romania, with a country-specific population, the primary objective of this research is to evaluate the longitudinal impact of pediatric seizures on family stress and coping mechanisms, from the initial ER visit through to long-term follow-up at 6 and 12 months. Additionally, the study will assess the effectiveness of integrated care models in supporting these families, focusing on psychological resilience and adaptation to the chronic nature of the disorder.

## 2. Materials and Methods

### 2.1. Study Design and Ethical Considerations

A longitudinal study was designed to evaluate the stress levels and coping mechanisms in families of children presenting with their first seizure episode and subsequent recurrent seizures within a 12-month period. This study adhered to the ethical standards outlined in the Declaration of Helsinki and received approval from the Ethical Commission of the involved institutions. Background information, medical history, and neurological diagnosis data were primarily extracted from hospital databases at the Emergency Clinical Hospital for Children “Louis Turcanu,” from Timisoara, Romania. To facilitate consistent and longitudinal data collection, follow-up assessments at 6 months and 1 year were scheduled, with some components administered online when necessary to accommodate family circumstances and ongoing medical advice. Families were recruited through the Emergency Clinical Hospital for Children ‘Louis Turcanu’, where children presenting with their first seizure episode were identified. Parents or legal guardians were approached for participation during their initial visit.

Inclusion criteria for this study are as follows: 1. Children aged between 1 and 18 years at the time of the initial emergency room presentation due to a seizure. 2. Children who have received a clinical diagnosis of a seizure disorder by a pediatric neurologist based on the emergency room assessment and subsequent evaluations. 3. Consent from parents or legal guardians for participation in the study, including follow-up assessments at 6 months and 1 year.

Exclusion criteria include: 1. Children who do not experience additional seizures within 12 months following their initial ER presentation. 2. Children with a known neurological condition that could confound the assessment of new-onset seizure disorders, such as significant traumatic brain injury, cerebral palsy, or other pre-existing severe neurological deficits. 3. Families who are unable or unwilling to commit to the follow-up schedule as outlined in the study protocol.

### 2.2. Questionnaires and Variables

The variables considered included the age and gender of the child, family’s area of residence, parental marital status, income level, education level, employment status, number of siblings in the household, and details of the seizure episodes such as frequency and severity. To comprehensively measure the psychological impact on the families, we utilized several validated questionnaires including the Parental Concerns Questionnaire (PCQ), the Hospital Anxiety and Depression Scale (HADS), the Perceived Stress Scale (PSS-10), and the Parenting Stress Index (PSI).

The HADS [22] is designed to identify levels of anxiety and depression, particularly suitable for hospital and outpatient settings. It consists of 14 items, which are split into two sections, with seven items focused on anxiety (HADS-A) and seven on depression (HADS-D), scored on a 4-point scale. Higher scores indicate greater distress, and it typically shows Cronbach’s alpha values ranging from 0.70 to 0.90.

The PSS-10 [23] is a 10-item scale that measures perceived stress, assessing how unpredictable, uncontrollable, and overloaded individuals perceive their life to be. The items are rated on a 5-point scale from 0 (“never”) to 4 (“very often”), with higher scores indicating greater perceived stress. The PSS-10 is noted for its strong psychometric properties with a Cronbach’s alpha typically above 0.70, reflecting its reliability.

The PSI [24] is designed to assess the stress levels experienced by parents, identifying specific sources of stress in the parent-child dynamics. It encompasses several domains and is scored on a 5-point scale, with higher scores indicating higher stress levels. The PSI is extensively validated with a Cronbach’s alpha generally above 0.85, underscoring its consistency and reliability in measuring parenting stress.

Finally, the PCQ [25] captures parents’ worries and concerns about their child’s health, development, and behavior. It is structured as a list of items that parents rate based on their level of concern, scored similarly on a scale where higher values indicate greater concerns. The PCQ is known for its practical application in pediatric assessments and typically exhibits a Cronbach’s alpha around 0.80, ensuring its reliability in capturing parental concerns accurately.

### 2.3. Statistical Analysis

Data management and analysis for this study were carried out using the statistical software SPSS version 26.0 (SPSS Inc., Chicago, IL, USA). The sample size was determined using a convenience sampling strategy, targeting a minimum of 62 participants to achieve a 95% confidence level with a margin of error of 10%. This was meant to achieve a power of 80% at a significance level of 0.05. Continuous variables were presented as mean ± standard deviation (SD), and categorical variables were described using frequencies and percentages. Student’s *t*-test was applied to compare two means, whereas the Analysis of Variance (ANOVA) test was employed to analyze differences among more than two means of continuous variables. For categorical data, the Chi-squared test was used. Statistical significance was established at a *p*-value of less than 0.05. All results underwent a double-check process to confirm accuracy and ensure the reliability of the findings.

## 3. Results

The study included a total of 68 patients who were followed for at least 12 months after their initial seizure. The average age of the children at initial presentation was 4.46 years (SD = 2.36), indicating a young cohort primarily in early childhood, with a significant majority (52.94%, n = 36) in the 6–12 year age range. Parents, with a mean age of 29.27 years (SD = 4.78), were relatively young, with most being married (73.53%, n = 50). The study’s population exhibited a diverse socioeconomic background, with half of the families falling into the middle income category (50.00%, n = 34), and a significant proportion having a college degree (51.47%, n = 35).

Gender distribution among the children showed a slight male predominance (55.88%, n = 38), which is consistent with the higher incidence of seizure disorders reported in male children in other epidemiological studies. In terms of family structure, a majority of families had one or two siblings (50.00%, n = 34), reflecting typical family sizes. The educational attainment of the parents was quite high, with over half holding at least a college degree, which could influence both the perception and management of their child’s health condition (Table 1).

In the evaluation of family coping mechanisms through the PSI, the study demonstrated a significant decrease in stress levels across multiple dimensions over the follow-up period. Initial assessments showed high levels of stress in categories such as Emotional Stress (59.47 ± 18.32), Parent–Child Communication Difficulties (66.03 ± 20.15), Behavioral Challenges in Child (62.11 ± 19.08), and Total Family Stress (65.55 ± 21.65). By the 6-month follow-up, there was a measurable reduction in stress scores, which continued to decrease by the one-year mark, with Emotional Stress reaching 50.63 ± 16.88, Parent–Child Communication Difficulties at 56.92 ± 18.74, Behavioral Challenges at 53.45 ± 17.93, and Total Family Stress at 55.97 ± 20.30. All values showed statistically significant improvements over time (*p*-value < 0.05).

**Figure 1:**
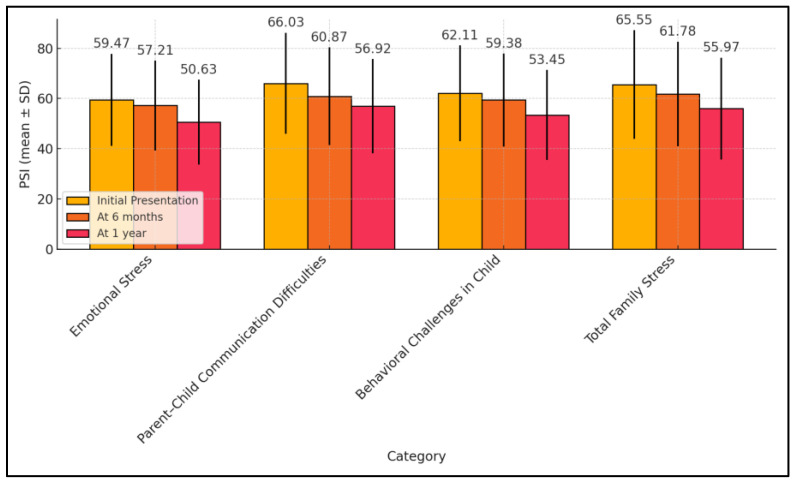
Follow-up of PSI survey results.

The analysis, performed using the ANOVA test, suggested that the interventions or natural adaptations occurring post-initial crisis might have been effective in reducing the overall stress experienced by families. The substantial drop in Total Family Stress, in particular, from 65.55 to 55.97, highlighted the potential for resilience and adaptation among these families. This decline in stress levels not only suggests improvements in the coping mechanisms but also underscores the importance of continued support and tailored interventions for families facing pediatric seizure disorders, promoting better outcomes in long-term family dynamics and child management (Table 2 and Figure 1).

The longitudinal assessment of emotional well-being using the HADS in families of children with seizure disorders indicated a significant improvement in mental health over the course of the study. Initially, the anxiety and depression scores were 8.2 ± 3.1 and 7.1 ± 2.8 respectively, reflecting a moderate level of psychological distress among the caregivers. By the six-month follow-up, these scores had modestly decreased to 7.5 ± 2.9 for anxiety and 6.8 ± 2.6 for depression. By the end of one year, further reductions were observed, with anxiety scores lowering to 6.8 ± 3.3 and depression scores to 5.9 ± 2.7. The *p*-values associated with these declines (0.033 for anxiety and 0.028 for depression) confirmed that the reductions were statistically significant.

**Figure 2:**
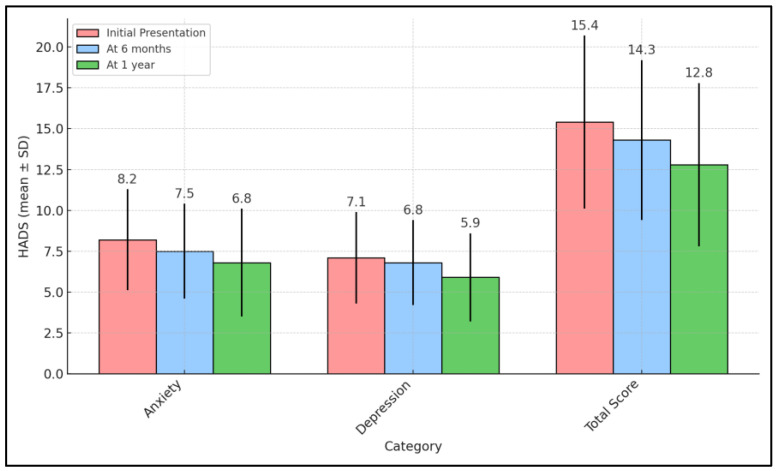
Follow-up of HADS survey results.

The overall HADS total score, combining both anxiety and depression dimensions, also showed a meaningful decrease, moving from an initial score of 15.4 ± 5.3 to 14.3 ± 4.9 at six months, and finally to 12.8 ± 5.0 by one year, with a *p*-value of 0.017. This trend suggested that as families adapted to managing their child’s seizure disorder, their cumulative burden of anxiety and depression lessened significantly. This reduction might be attributed to a combination of factors including increased familiarity with the condition, improved coping strategies, and possibly the effect of the interventions or support received during the study period (Table 3 and Figure 2).

**Figure 3:**
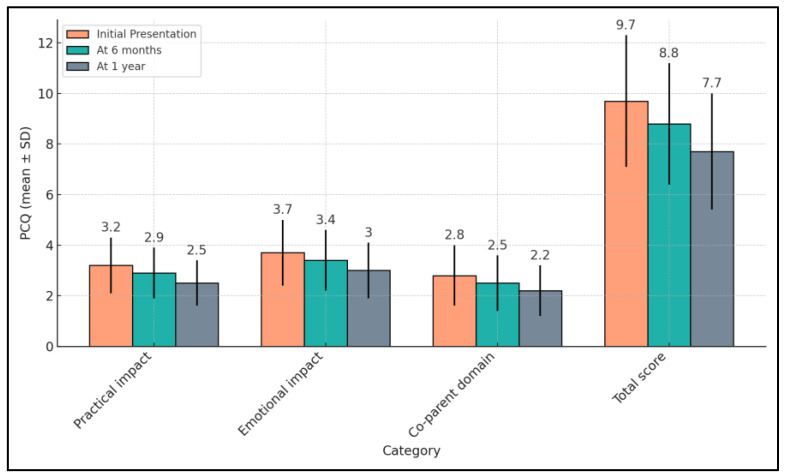
Follow-up of PCQ survey results.

In the study examining family coping mechanisms in pediatric seizure disorders, the analysis of the PCQ indicated noteworthy reductions in parental concerns over a one-year period. Initially, concerns in the practical impact domain started at a level of 3.2, which decreased to 2.9 at six months and further reduced to 2.5 by the one-year mark. The emotional impact showed a similar downward trend, beginning at 3.7, decreasing to 3.4 at the six-month follow-up, and reaching 3.0 after one year. Concerns in the co-parent domain also decreased over time, starting from 2.8, reducing to 2.5 at six months, and finally to 2.2 at one year. The total score, summarizing overall parental concerns, followed this pattern as well, starting at 9.7 and decreasing progressively to 8.8 and then 7.7 at six and twelve months, respectively. These results reflect a significant alleviation in parental concerns across all domains, illustrating a positive shift in the coping mechanisms as families adapted to managing their child’s seizure condition (Table 4 and Figure 3).

**Figure 4:**
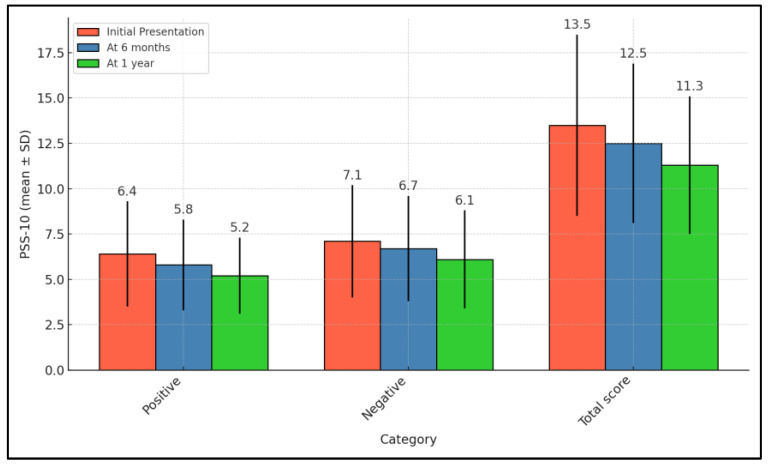
Follow-up of PSS-10 survey results.

The PSS-10 results highlighted a gradual decrease in perceived stress among the participating families. The initial presentation recorded a total PSS-10 score of 13.5, indicating a considerable level of stress, which decreased consistently to 12.5 at the six-month follow-up and further to 11.3 by the end of the year. This trend was statistically significant, as indicated by a *p*-value of 0.016. Specifically, the positive aspects of stress reduced from an initial score of 6.4 to 5.8 at six months to 5.2 at one year, also showing significant improvement with a *p*-value of 0.023. However, the reduction in the negative aspects of stress did not reach statistical significance, with scores starting at 7.1 and modestly decreasing to 6.7 and 6.1 at six months and one year, respectively (*p*-value of 0.123), as seen in Table 5 and Figure 4.

## 4. Discussion

### 4.1. Literature Analysis

The current study provided significant insights into the effects of multidisciplinary follow-up care on reducing stress and enhancing coping strategies in families dealing with pediatric seizure disorders. Results demonstrated a statistically significant reduction in overall perceived stress as measured by the PSS-10, with a marked decrease in both the positive and negative stress scores over the course of the year. Initially, families reported a total PSS-10 score of 13.5, which notably reduced to 11.3 by the year’s end. This reduction suggests that families gradually adapted to the stressors associated with managing a pediatric seizure disorder.

Interestingly, while the overall stress levels decreased, the reductions in negative stress did not achieve statistical significance, hinting at persistent underlying stressors that may not have been fully addressed by the interventions or natural adaptations. This underscores the complexity of stress dynamics within families dealing with chronic health issues, where positive stress reduction is more readily achieved than negative. This aspect of the findings highlights the need for targeted interventions that specifically address the more resistant elements of stress and suggests further refinement of support mechanisms to better serve these families.

Furthermore, the significant improvements in the PSI across various domains including emotional stress, parent–child communication difficulties, and behavioral challenges underscore the efficacy of ongoing support and intervention. This broad improvement across multiple stress-related domains indicates that comprehensive care approaches, possibly involving psychological support and educational programs, are effective in helping families adjust to the demands of managing a chronic pediatric condition.

In two separate studies, researchers investigated the impact of epilepsy on the quality of life (QoL) in children and the extended effects on their families. Rozensztrauch and Kołtuniuk [26] conducted a cross-sectional survey involving 103 legal guardians, using the PedsQL 4.0 and PedsQL 2.0 Family Impact Module. Their findings highlighted a considerable decrement in family activities and relationships, with an overall family activities score averaging 32.4 and relationships at 55.63. The study further indicated that younger children aged 5–7 years experienced a lower QoL compared to those aged 2–4 years, by approximately 11.956 points. Similarly, the study by Subki et al. [27] involved 80 mothers using the “Impact of Pediatric Epilepsy Scale” (IPES) and identified that the average child’s QoL was markedly low at 2.85, with 87.5% of the mothers rating their child’s QoL as low. The findings also revealed that the QoL and impact of epilepsy were significantly associated with seizure frequency and the child’s nationality, suggesting a nuanced influence of cultural and medical factors on the child’s well-being.

In exploring the multifaceted impact of pediatric epilepsy on families, two studies offer insightful findings with measurable outcomes. Camfield et al. [28] developed an 11-item scale, the Impact of Pediatric Epilepsy Scale (IPES), which parents used to rate the psychosocial effects of epilepsy on their children and family life. With a high internal validation (Cronbach’s alpha of 0.92), the study demonstrated that a higher IPES score, indicative of more severe impacts of epilepsy, correlated with increased parental stress, lower sibling respect, reduced self-esteem, and heightened emotional problems in the children. Moreover, this total impact was significantly related to clinical factors like seizure frequency and medication load. Similarly, the study by Nagabushana et al. [29] assessed the quality of life (QoL) of children with epilepsy in India using the Quality of Life in Children with Epilepsy (QOLCE) questionnaire. Their findings pointed out that children experiencing more severe seizures, those on multiple antiepileptic drugs (AEDs), and those with poor adherence to medication schedules showed significantly lower QoL scores. Notably, children manifesting adverse effects from AEDs had markedly lower scores across all QoL domains.

In the study by Choong Yi Fong et al. [30], the Quality of Life Measurement for Children with Epilepsy (CHEQOL-25) was utilized to capture both child and parent perceptions of QoL. This study found that parents generally rated their children’s QoL lower than the children did themselves, with a mean parent score of 68.56 versus a child score of 71.82. Notably, the agreement between child and parent assessments was poor to moderate, particularly in the domains related to epilepsy secrecy, suggesting a divergence in perception primarily influenced by the social stigma associated with epilepsy. Malay ethnicity, focal seizures, and high seizure frequency were identified as factors associated with lower CHEQOL-25 scores, indicating specific demographic and clinical attributes that may necessitate targeted interventions. In a similar manner, the study conducted by Su Woan Wo et al. [31] focused on the cross-cultural adaptation and validation of the Malay version of the parent-proxy, CHEQOL-25. This adaptation was found to be both valid and reliable, with Cronbach’s alpha values demonstrating strong internal consistency and good test–retest reliability. Furthermore, the severity of epilepsy, the number of antiepileptic drugs, and the child’s social interactions were significantly linked to lower QoL scores.

The Canadian study by Verhey et al. [32] utilized the CHEQOL-25 to compare self-reported and proxy-reported QoL among 375 children with active epilepsy and their 378 parents, revealing an overall modest intraclass correlation coefficient of 0.45, indicating moderate agreement between child and parent perspectives. Notably, the greatest discrepancies were observed in the more subjective subscales such as Secrecy and Present Concerns. Additionally, factors like the child’s age and school placement appeared to influence these discrepancies, suggesting that as children grow older or depending on their educational context, their self-perception of QoL diverges more from their parents’ perceptions. In a similar manner, the study led by Stevanovic et al. [33], in Serbia, a neighboring country with Romania, involved translating and adapting the CHEQOL-25 for Serbian children and their parents, and also reported a moderate level of agreement between children and parents, with correlation coefficients ranging from 0.43 to 0.57 across various subscales. This study found that while most subscales demonstrated sufficient reliability, there was a notable variation in internal consistency, indicating some challenges in the measurement’s reliability across different contexts.

The multidisciplinary follow-up care implemented in our study involved a comprehensive support system tailored specifically for families of children with pediatric seizure disorders. This included regular consultations with pediatric neurologists, access to counseling services from child psychologists, and continuous education and support from specialized nurses and social workers. This integrated approach was designed to address not only the medical needs of the child but also the emotional and psychological well-being of the entire family unit throughout the critical first year following the initial seizure episode.

The data thus support the hypothesis that proactive, multidisciplinary follow-up care can significantly alleviate stress and improve coping mechanisms in families of children with pediatric seizure disorders. The findings advocate for the continuation and expansion of integrated care models that not only address medical needs but also focus on psychological resilience and family dynamics, which are crucial for long-term adaptation to chronic pediatric health conditions. This approach could potentially be mirrored in other pediatric chronic disease management strategies to enhance overall family well-being.

### 4.2. Strengths and Limitations

The study does have limitations that must be considered. Its design, relying on convenience sampling and a relatively small sample size, may limit the generalizability of the findings. The study’s population, drawn from a single hospital, may not reflect the wider diversity of socioeconomic backgrounds and healthcare settings that exist globally. Additionally, the self-reported nature of the questionnaires might introduce bias in the responses, with participants potentially underreporting or overreporting their stress levels due to social desirability. The sampling procedure’s primary limitation lies in its reliance on convenience sampling and the surveying of only one parent per family, typically the one more present during medical appointments. This approach may introduce selectivity bias and limit the validity of the findings, as it does not capture the perspective of both partners. These factors may affect the generalizability of the results to all families dealing with pediatric seizure disorders.

The absence of a control group is indeed a limitation of our study as it restricts our ability to definitively attribute the observed improvements in stress, anxiety, and coping mechanisms directly to the multidisciplinary care provided. Future research could enhance our findings by including a control group of families who receive standard care, allowing for a more rigorous assessment of the specific impact of multidisciplinary follow-up care on these families’ adaptation and stress reduction over time.

The longitudinal design of this study, despite its inherent challenges, provided a unique perspective on the temporal dynamics of stress and coping in families dealing with pediatric seizures. This approach allowed us to capture the gradual changes in psychological well-being and stress management, underscoring the effectiveness of multidisciplinary care. Future studies could benefit from our methodology by incorporating similar strategies to mitigate sample attrition and data management challenges, further enriching our understanding of chronic conditions in pediatric populations.

## 5. Conclusions

In conclusion, the current study effectively demonstrates that a comprehensive, multidisciplinary follow-up care approach can significantly alleviate family stress and enhance coping mechanisms in the context of pediatric seizure management. These findings highlight the importance of integrated care models that go beyond medical treatment to address the psychological and emotional needs of families, advocating for broader implementation of such models in pediatric healthcare. The study’s implications point toward a more holistic approach to chronic pediatric health conditions, emphasizing the critical role of psychological and social support in achieving favorable outcomes.

## Figures and Tables

**Table 1:** Background characteristics of studied patients.

Variables	n = 68	%
Age of Child (mean ± SD)	4.46 years	2.36
Age of Parent (mean ± SD)	29.27 years	4.78
Child Age Range: 0–5 years (%)	12	17.65%
Child Age Range: 6–12 years (%)	36	52.94%
Child Age Range: 13–18 years (%)	20	29.41%
Gender: Male (%)	38	55.88%
Gender: Female (%)	30	44.12%
Family Income Level: Low (%)	14	20.59%
Family Income Level: Middle (%)	34	50.00%
Family Income Level: High (%)	20	29.41%
Parental Marital Status: Married (%)	50	73.53%
Parental Marital Status: Other (%)	18	26.47%
Education Level (Parent): High School or Less (%)	15	22.06%
Education Level (Parent): College Degree (%)	35	51.47%
Education Level (Parent): Advanced Degree (%)	18	26.47%
Number of Siblings: None (%)	22	32.35%
Number of Siblings: 1 or 2 (%)	34	50.00%
Number of Siblings: 3 or more (%)	12	17.65%

SD—Standard Deviation.

**Table 2:** Follow-up of PSI survey results.

PSI (Mean ± SD)	Initial Presentation (n = 68)	At 6 Months (n = 68)	At 1 Year (n = 68)	*p*-Value *
Emotional Stress	59.47 ± 18.32	57.21 ± 17.89	50.63 ± 16.88	0.011
Parent–Child Communication Difficulties	66.03 ± 20.15	60.87 ± 19.46	56.92 ± 18.74	0.025
Behavioral Challenges in Child	62.11 ± 19.08	59.38 ± 18.55	53.45 ± 17.93	0.022
Total Family Stress	65.55 ± 21.65	61.78 ± 20.84	55.97 ± 20.30	0.029

* ANOVA test; SD—Standard Deviation; PSI—Parental Stress Index (Higher scores on the PSI indicate greater levels of stress).

**Table 3:** Follow-up of HADS survey results.

HADS (Mean ± SD)	Initial Presentation (n = 68)	At 6 Months (n = 68)	At 1 Year (n = 68)	p-Value *
Anxiety	8.2 ± 3.1	7.5 ± 2.9	6.8 ± 3.3	0.033
Depression	7.1 ± 2.8	6.8 ± 2.6	5.9 ± 2.7	0.028
Total Score	15.4 ± 5.3	14.3 ± 4.9	12.8 ± 5.0	0.017

* ANOVA test; SD—Standard Deviation; HADS—Hospital Anxiety and Depression Scale (higher scores indicate greater levels of anxiety or depression).

**Table 4:** Follow-up of PCQ survey results.

PCQ (Mean ± SD)	Initial Presentation (n = 68)	At 6 Months (n = 68)	At 1 Year (n = 68)	p-Value *
Practical impact	3.2 ± 1.1	2.9 ± 1.0	2.5 ± 0.9	<0.001
Emotional impact	3.7 ± 1.3	3.4 ± 1.2	3.0 ± 1.1	0.004
Co-parent domain	2.8 ± 1.2	2.5 ± 1.1	2.2 ± 1.0	0.007
Total score	9.7 ± 2.6	8.8 ± 2.4	7.7 ± 2.3	<0.001

* ANOVA test; SD—Standard Deviation; PCQ—Parental Concerns Questionnaire (higher scores indicate greater levels of concern or anxiety among parents).

**Table 5:** Follow-up of PSS-10 survey results.

PSS-10 (Mean ± SD)	Initial Presentation (n = 68)	At 6 Months (n = 68)	At 1 Year (n = 68)	*p*-Value *
Positive	6.4 ± 2.9	5.8 ± 2.5	5.2 ± 2.1	0.023
Negative	7.1 ± 3.1	6.7 ± 2.9	6.1 ± 2.7	0.123
Total score	13.5 ± 5.0	12.5 ± 4.4	11.3 ± 3.8	0.016

* ANOVA test; SD—Standard Deviation; PSS-10—Perceived Stress Scale (higher scores indicate higher levels of perceived stress).

## Data Availability

The data presented in this study are available on request from the corresponding author.
